# Hyper-Arousal Decreases Human Visual Thresholds

**DOI:** 10.1371/journal.pone.0061415

**Published:** 2013-04-08

**Authors:** Adam J. Woods, John W. Philbeck, Philip Wirtz

**Affiliations:** 1 Center for Functional Neuroimaging, Department of Neurology, University of Pennsylvania, Philadelphia, Pennsylvania, United States of America; 2 Center for Cognitive Neuroscience, University of Pennsylvania, Philadelphia, Pennsylvania, United States of America; 3 Department of Psychology, The George Washington University, Washington, DC, United States of America; 4 Department of Decision Sciences, The George Washington University, Washington, DC, United States of America; Colorado State Univeresity, United States of America

## Abstract

Arousal has long been known to influence behavior and serves as an underlying component of cognition and consciousness. However, the consequences of hyper-arousal for visual perception remain unclear. The present study evaluates the impact of hyper-arousal on two aspects of visual sensitivity: visual stereoacuity and contrast thresholds. Sixty-eight participants participated in two experiments. Thirty-four participants were randomly divided into two groups in each experiment: Arousal Stimulation or Sham Control. The Arousal Stimulation group underwent a 50-second cold pressor stimulation (immersing the foot in 0–2° C water), a technique known to increase arousal. In contrast, the Sham Control group immersed their foot in room temperature water. Stereoacuity thresholds (Experiment 1) and contrast thresholds (Experiment 2) were measured before and after stimulation. The Arousal Stimulation groups demonstrated significantly lower stereoacuity and contrast thresholds following cold pressor stimulation, whereas the Sham Control groups showed no difference in thresholds. These results provide the first evidence that hyper-arousal from sensory stimulation can lower visual thresholds. Hyper-arousal's ability to decrease visual thresholds has important implications for survival, sports, and everyday life.

## Introduction

Conscious sensory perception is dependent upon the coordinated activity of arousal and sensory systems in the brain. [1] Arousal serves as a substrate of consciousness that is a) important for providing a basic aptitude for behavioral response to the environment and b) a means for optimizing behavior. [2]–[5] Just as hypo-arousal can compromise our ability to respond to the environment (e.g., coma), hyper-arousal holds the potential to enhance our performance (e.g., fight or flight). [4]–[11] In most domains of vision science, however, arousal is not incorporated into theoretical models. Arousal fluctuates naturally over the course of the day, and many activities, social situations, foods, and health factors can influence arousal. If arousal does play a role in modulating visual perception, many current models fail to capture a nearly ubiquitous source of variation. Thus, there is a pressing need to investigate the possible consequences of increased arousal for visual perception.

The present study begins an investigation of the influence of hyper-arousal on perceptual judgments of visual information in the environment. Hyper-arousal is any increase in arousal above an organism's normal awake arousal state. [3], [5] Normal arousal, or normo-arousal, is the average level of arousal in an awake, non-brain damaged organism and serves as the baseline state of responsiveness for conscious behavior. [1], [2] Hyper-arousal can range from small increases due to caffeine intake or startle response to abnormally high levels associated with post-traumatic stress disorder or high-voltage electric shock. [5], [12] In particular, the present study investigates whether inducing a state of hyper-arousal through sensory stimulation improves the discriminability of 1) depth relationships signaled by binocular disparity and 2) contrast gratings.

Visual space perception is a particularly crucial perceptual domain, in that it allows us to represent the shape and layout of things in the environment, interact effectively with people and other objects, and plan future spatial behaviors. Furthermore, sensitivity to contrast provides us with information about the boundaries of objects, facilitates object identification, and guides attentional prioritization. Arousal-based improvement in depth and contrast processing would have obvious implications for survival. In situations involving immediate threats in the environment, increased appreciation of depth could prove essential for avoiding obstacles, localizing targets for reaching or grasping actions, or planning routes through the environment. Increased discrimination of contrast could prove critical for threat detection and accurate representation of obstacle boundaries. Even in less dire situations, such improvement in depth and contrast processing would potentially benefit performance in sports, driving, and many other contexts.

A particularly well-studied means of increasing arousal is cold pressor stimulation (CPS). CPS administration of 1–2 min is commonly used in clinical evaluations of autonomic nervous system function and as a cardiovascular response test. [13]–[17] At longer durations (e.g., 4–5 min), it has also been used in studies of pain threshold and tolerance. [18] Stimulation triggers sympathetic activation leading to vasoconstriction. [15] Heart rate and blood pressure are normally elevated within the first minute of CPS and then return to baseline minutes after stimulation ends. [13]–[17] This response is reliable and demonstrates minimal attenuation when re-tested 2 weeks later. [19] Functional magnetic resonance imaging studies also suggest that CPS activates a wide range of cortical and subcortical structures in the brain, including: the lateral and inferior postcentral gyrus; aspects of the inferior, middle, and superior frontal gyri; anterior insula; anterior cingulate gyrus; occipital and temporal cortices; the thalamus; the anterior and posterior hypothalamus; amygdala; hippocampus; cerebellar cortex; and pontine areas. [20]–[23] This wide range neural activation is consistent with the broad pattern of effect expected with change in arousal.

In the present study, we used a 50 second CPS to induce a hyper-arousal response. Previous electrophysiological research demonstrated that immersing the foot for 50 seconds in 0–2° C water results in a state of hyper-arousal lasting for approximately ten minutes. [3], [4] For example, using the P50 evoked-response potential, a marker of ascending reticular activating system (ARAS) output, Woods et al (2011) demonstrated that CPS induces an arousal regulation response in healthy participants. [4] Specifically, the amplitude of the P50 ERP increased or decreased relative to a person's initial state of arousal. That is to say, if a person is already hyper-aroused at the time of stimulation, arousal-related output of the ARAS is downregulated, thus tending to prevent a state of hyper-arousal that might otherwise be detrimental for behavioral performance, and vice versa for a person in a state of hypo-arousal. These results, in addition to other recent findings, suggest that regulation of arousal output is likely mediated by brain systems exerting regulatory control over the ARAS and serves to promote an optimal state for responding to the surrounding environment. [24]–[28]

Recent animal research demonstrates that arousal states modulate the responsiveness of neurons in the early visual system. [29]–[30] These effects occur as early as the lateral geniculate nucleus, before information reaches the visual cortex. Thus, although rarely considered in theories of higher-level visual perception, arousal has strong implications for the vision sciences. Unfortunately, the mechanisms behind arousal-related improvement in response to the environment remain unclear. Previous research demonstrates that transient exogenous attentional cuing to a particular spatial location or object can enhance the visual appearance of contrast at the cued location and lower visual contrast thresholds there. [31]–[33] Hyper-arousal-related improvements may also function through facilitation of attentional mechanisms. Arousal and attention have long been known to share a reciprocal relationship. For example, while states of hypo- and hyper-arousal modulate attentional processes, sustained and focused attentional processes modulate arousal state in tasks requiring sustained performance. [3], [34]–[37] In a similar vein, recent research demonstrates that emotionally arousing visual stimuli (e.g., fearful faces) gain preferential access to awareness, predominate over less arousing stimuli, and selectively impact different aspects of low-level contrast sensitivity. [38]–[40] These data collectively suggest that transient increases in emotional arousal provide an advantage for processing certain arousing visual stimuli in the environment. Recent research by Phelps et al (2006) suggests that effects of fearful faces on low-level vision can be explained, at least in part, by attentional mechanisms. [35]

However, unlike transient emotional arousal or attentional cueing manipulations, CPS-induced hyper-arousal responses are sustained and generalized (i.e., lasting approximately 10 minutes and irrespective of the side of the body stimulated). [3], [4] Significant effects from a sustained and generalized arousal manipulation would be an important discovery on several fronts. As many factors can result in sustained changes in arousal, arousal-related effects on visual thresholds could broadly impact survival, sports performance, and everyday life. Furthermore, significant decreases in visual thresholds using a sustained and generalized manipulation, rather than a transient spatial cue, would provide an important foundation for future investigations into the role of attention in arousal-related effects. Finally, significant effects of arousal on visual thresholds would provide evidence for the importance of including this factor in models of visual perception.

To assess the impact of hyper-arousal on human visual thresholds, we measured stereoacuity and contrast thresholds before and immediately after CPS in the current study. There are three plausible patterns of result in the current study: 1) both stereoacuity and contrast thresholds will decrease, 2) only one of the two thresholds will decrease, or 3) neither of the two thresholds will decrease following a CPS-induced hyper-arousal response. The first pattern of result would suggest that the role of arousal in visual thresholds is not restricted to a single visual domain, but instead spans multiple domains. In contrast, the second pattern would suggest that the role of arousal is more limited in scope. In contrast, the last pattern of result would suggest that there is likely limited benefit in accounting for arousal states in models of visual perception. A fourth, albeit unlikely, possible outcome is that CPS could lead to a *decrement* in performance. This might happen if CPS were to induce an extreme state of hyper-arousal. [5] However, past work suggests that 50 sec of CPS stimulation does not produce the kind of extreme hyper-arousal associated with performance decrements, but rather an arousal regulation response. [3]–[4]

## Experiment 1: Stereoacuity

Experiment 1 investigated whether a CPS induced hyper-arousal response can increase people's ability to discriminate subtle depth relationships signaled by binocular disparity (i.e., lower thresholds).

### Materials and Methods

#### Ethics statement

The study was approved by the George Washington University Institutional Review Board. All participants in the present research (n = 68) gave written informed consent.

#### Subjects

Participants were 34 college age volunteers who received course credit for participation. Participants were randomly divided into either an Arousal Stimulation Group (n = 17, mean age±SD = 19.3±1.0 years, 12 females) or a Sham Control Group (n = 17, mean age±SD = 19.4±0.8 years, 11 females). All participants were naïve to the purpose of the study and reported normal or corrected to normal vision.

#### Design

The experiment took place in a well-lit indoor classroom. Participants in the Arousal Stimulation Group underwent CPS-immersing the foot for 50 seconds in 0–2° C water. Rather than performing CPS, participants in the Sham Control Group underwent a “sham” stimulation-immersing the foot in room temperature water (22–24° C) for 50 seconds. [4] Neither group was aware of the opposing group. Participants in a given group only received one form of stimulation (i.e., CPS or Sham) and underwent depth threshold testing before (Baseline) and after (Post-Stimulation) the appropriate stimulation procedure. A between subjects design was chosen to minimize engagement of demand characteristics associated with conscious knowledge of experimental manipulations in experiments and focus results on relative changes associated with different forms of stimulation.

Participants in both groups received the same instructions. The experimenter (AJW) used a neutral affect and followed a scripted conversation with participants in both groups. A scripted conversation was used so that the CPS or Sham stimulation were always referred to consistently between subjects and never discussed using language that referred to arousal, cold water, etc. Participants first underwent practice trials, followed by Baseline test trials. Following Baseline testing, participants underwent the CPS or Sham stimulation for 50 seconds. Immediately following stimulation, participants underwent a final set of Post-Stimulation test trials. Side of stimulation (i.e., left or right foot) was counterbalanced across participants.

#### Cold pressor and sham apparatus

CPS was prepared in a closable insulated cooler measuring 14 inches by 10 inches. Equal volumes of water and ice were placed in the cooler. A digital aquarium thermometer was attached below the water line to allow monitoring of water temperature. CPS was prepared 15 minutes prior to the participant's arrival and allowed to attain the targeted temperature between 0 and 2 degrees Celsius. Sham stimulation was prepared 1 hour prior to participant arrival using the same cooler but water was added and allowed to sit with the top open until the targeted 22–24 degrees range was attained. Targeted temperatures could be maintained for over one hour once the cooler was closed.

#### Stereoscopic depth apparatus

**Figure 1:**
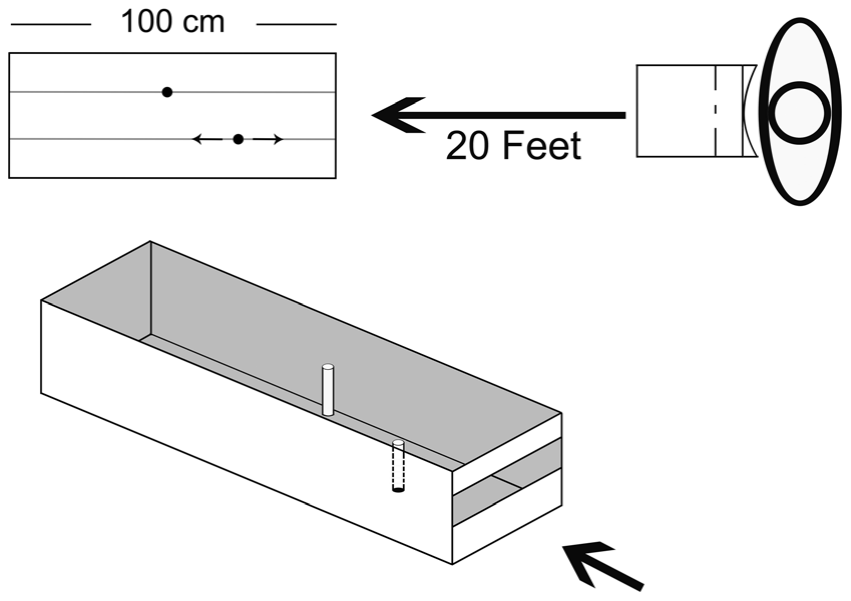
Depth threshold device. Participants viewed two white rods through an occluder placed at optical infinity (20 feet). Participants judged which rod (left or right) was closer in depth to their location. The maximum depth interval was 50 cm.

Stereoscopic depth threshold was measured using a two-alternative forced choice technique. The task required participants to indicate which of two rods was closer in depth to their location (i.e., left or right). The target holder was a wooden box mounted on a tripod. The target holder was constructed such that the participant observed two white rods (5 cm×0.7 cm) at various intervals of depth (Figure 1). The rods had a 2.5 cm separation and the left-most rod was stationary. The right-most rod was placed on a hidden slider that allowed it to be adjusted along a range of 100 cm with different test depth intervals ranging from 50 cm (or approximately 140 arcsec of disparity) in front or behind the stationary rod. All surfaces on the target holder were painted matte black to present a consistent texture. The target holder was placed 20 feet from the participant's viewing location to minimize the influence of egocentric distance cues, leaving binocular disparity as the primary stimulus cue to the relative depth between the rods. Participants viewed the target holder through a table-mounted occluder with adjustable chin-rest allowing binocular viewing of the target holder at eye-level.

The table-mounted occluder allowed participants to place their face in a molded viewing aperture contoured to fit the forehead and nose firmly in place, with eye-height maintained via the chin-rest. The occluder had a card slot that provided full occlusion of the environment between trials. The card could be removed by the participant at the beginning of each trial to view the target holder. The target holder was built to provide viewing of the two rods at a height of 110 cm with the front “open” surface built to occlude the top and bottom surface of the target rods. Thus, in addition to the disparity between the rods, a potentially useful disparity signal was also present between the occluder and each of the rods. This was equally true in all conditions, however, and thus did not impact our ability to test our primary research question, which hinged on group differences relative to their own baseline performance.

We used a variant of the QUEST adaptive threshold-seeking algorithm written by Denis Pelli for the Psychtoolbox in MATLAB (The Mathworks, Natick, MA). [41]–[43] On each trial, the experimenter entered the accuracy of a participant's response and the program calculated the optimal size of the next depth interval to be presented. The size of the depth interval varied adaptively from trial to trial based on the participant's prior responses. Side of depth (e.g., left rod closer or vice versa) was randomized across trials. Participants underwent a total of 40 trials. The stereoscopic depth threshold was defined as the linear separation between the rods along the line of sight that yielded 82 percent correct performance across the 40 trials.

#### Procedure

Prior to testing, the chin-rest on the table-mounted occluder was adjusted so that viewing height was 110 cm. Participants received instructions to respond either “Left” or “Right” indicating which of the two rods was closer to their eyes. Participants donned foam earplugs and tight fitting over-ear hearing protectors to remove any auditory cues generated by moving the rod in the depth apparatus between trials (i.e., a slight scraping noise that could provide temporal feedback on relative changes to rod depth between trials). Next, 6 practice trials were conducted. Practice trials were given at depth intervals of±8,±21, and±45 cm of separation in random order (approximately 23.8, 61.3, and 127 arcsec of separation; ± refers to the side of greatest depth, +8 = Left rod 8 cm farther than the right rod, −8 = Left rod 8 cm closer than the right rod). The practice trials were intended to accustom participants with the testing procedure. No error feedback was given.

In both the practice and experimental trials, participants kept their head located in the table-mounted occluder device. At the start of each trial, a card occluding the testing environment was put in place. The experimenters prepared the stimulus by placing the moveable rod at the proper location on the target box. When ready, the experimenter verbally signaled the participant to raise the occlusion card and look through the occluder. Participants verbally indicated which rod appeared closer to their location (i.e., Left or Right). After the response, the experimenter entered the participant's response into the computer and cued the participant to lower the occlusion card for the next trial.

#### Analyses

Data (depth thresholds) were evaluated performing a 2 (Group: Arousal Stimulation vs. Sham Stimulation) x 2 (Block: Baseline vs. Post-Stimulation) RM-ANOVA. Evidence of a significant Group x Block interaction would suggest at least one of the two groups evidenced a significant effect of Stimulation. Planned comparisons were paired samples t-tests. Numerical differences at baseline between groups were checked for significance using an independent t-test. Depth was analyzed in units of linear separation (cm), but approximate values in arcsec of separation are also provided (assuming an average interpupillary distance, IPD, of 6.3 cm).

### Results

**Figure 2:**
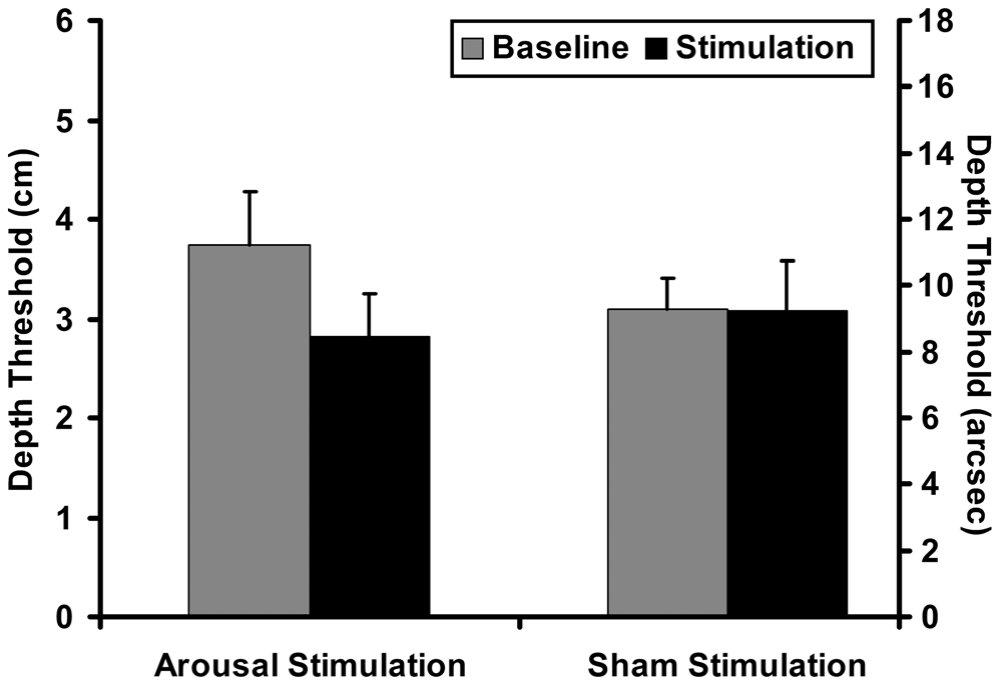
Stereoacuity thresholds. Mean stereoacuity threshold from Baseline to Post-Stimulation for the Arousal Group and Sham Control Group. The left y-axis reports thresholds in units of linear separation (cm). The right y-axis reports thresholds in approximate units of angular separation (arcsec). Arcsec was calculated using an assumed average IPD of 6.3 cm. Baseline performance was not significantly different between groups (t = 1.02; DF = 32; p = .31). * = p<.05

**Figure 3:**
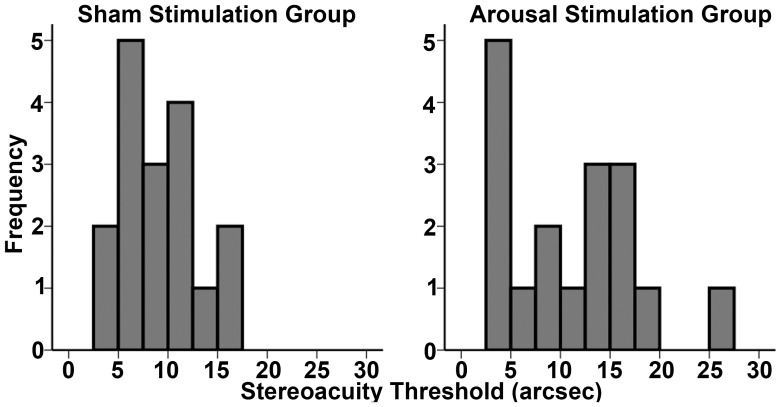
Frequency plots for baseline stereoacuity thresholds by group.

The RM-ANOVA demonstrated a significant Group x Block interaction (F_(1,16)_ = 4.66, Mean Square Error (MSE) = 3.4, p = 0.04, Partial Eta Squared (ηp2) = 0.27). Neither Group (F_(1,16)_ = 0.12, MSE = 0.6, p = 0.73) nor Block (F_(1,16)_ = 3.49, MSE = 3.7, p = 0.08) demonstrated a significant main effect. Planned paired samples t-tests of depth thresholds in the Arousal Stimulation group demonstrated a significant effect of arousal stimulation on stereoacuity thresholds (t = 3.71, DF = 16, p = 0.002, Cohen's *d* = 1.35; Figure 2) from baseline (mean threshold±standard error (M±SE) = 3.73±0.54 cm; ≈11.17±1.6 arcsec) to post-stimulation (M±SE = 2.81±0.54 cm; ≈8.42±1.3 arcsec). However, Sham stimulation had no effect on depth thresholds from baseline (M±SE = 3.09±0.31 cm; ≈9.27±0.9 arcsec) to post-stimulation (M±SE = 3.07±0.51 cm; ≈9.20±1.5 arcsec; t = 0.05, DF = 16, p = 0.9). There was no significant difference between baseline thresholds in the Arousal versus Sham stimulation groups (independent t-test: t = 1.02; DF = 32; p = .31). Thus, although slightly numerically different at baseline, there was not a significant difference between groups in terms of baseline stereoacuity thresholds (Figure 2). Nevertheless, frequency plots of baseline thresholds for the Arousal and Sham Stimulation Groups demonstrate some differences in the distribution of threshold values at baseline (Figure 3). To determine whether non-significant differences at baseline between groups account for significant effects of Arousal Stimulation, we performed an analysis of covariance (ANCOVA) with signed difference in threshold (post-stimulation - baseline) as the dependent variable with Group and Baseline as independent factors. When controlling for differences in baseline thresholds between groups, there remained a significant effect of Group (F_(1,30)_ = 4.4, MSE = 7.6, p = .04). Thus, numerical differences at baseline between Arousal and Sham Stimulation groups do not explain significant effects of CPS on stereoacuity thresholds.

### Discussion

Hyper-arousal from CPS sensory stimulation increased participants' sensitivity to subtle depth differences. However, sham stimulation had no influence on stereoacuity. These results suggest that sensitivity to at least some subtle visual differences in the environment is facilitated by changes in arousal state. However, it remains unclear whether these effects are specific to stereoacuity or generalize to other aspects of human visual perception. If arousal effects generalize to other aspects of visual perception, this would provide strong evidence for the importance of accounting for this nearly ubiquitous source of variation in models of visual perception.

## Experiment 2: Contrast

Experiment 2 investigated whether hyper-arousal effects generalize to another aspect of visual perception: contrast discrimination. As discussed earlier, consistent effects across stereoacuity and contrast would suggest that hyper-arousal has a relatively broad impact on detection of subtle visual differences in the environment. This pattern of result would provide strong evidence supporting future investigations into the role of arousal in other aspects of visual perception. Furthermore, this result would suggest that a sustained and non-spatial manipulation of arousal produces effects on visual thresholds similar to those previously shown in studies using transient attentional cueing to spatial locations. In contrast, lack of hyper-arousal related effects on contrast discrimination would suggest that the role of arousal is more limited in scope and may not broadly impact detection of subtle visual differences in the environment.

### Materials and Methods

#### Subjects

Participants for this study were 34 college age volunteers who received course credit for participation in the study. Participants were divided into either an Arousal Stimulation Group (n = 17, mean age ± SD = 19.3±1.0 years, 12 females) or a Sham Stimulation Control Group (n = 17, mean age±SD = 19.4±0.8 years, 11 females). All participants were naïve to the purpose of the study and reported normal or corrected to normal vision.

#### Design, cold pressor, and sham apparatus

CPS and Sham stimulation procedures and the pre-post testing design were identical to Experiment 1.

#### Contrast discrimination apparatus and stimuli

Contrast threshold was measured using a two-alternative forced choice task. The program was modified from an open access program (QuestDemo) written by Denis Pelli for the Psychtoolbox in MATLAB (The Mathworks, Natick, MA) on a Macintosh 2GHz PowerPC G5. [41]–[43] Screen resolution was 1440×900 with graphics acceleration via an ATI Radeon 9600 128MB graphics card. The program used the well-documented QuestMean procedure to determine trial-by-trial levels of contrast discriminability for stimuli. [44]

**Figure 4:**
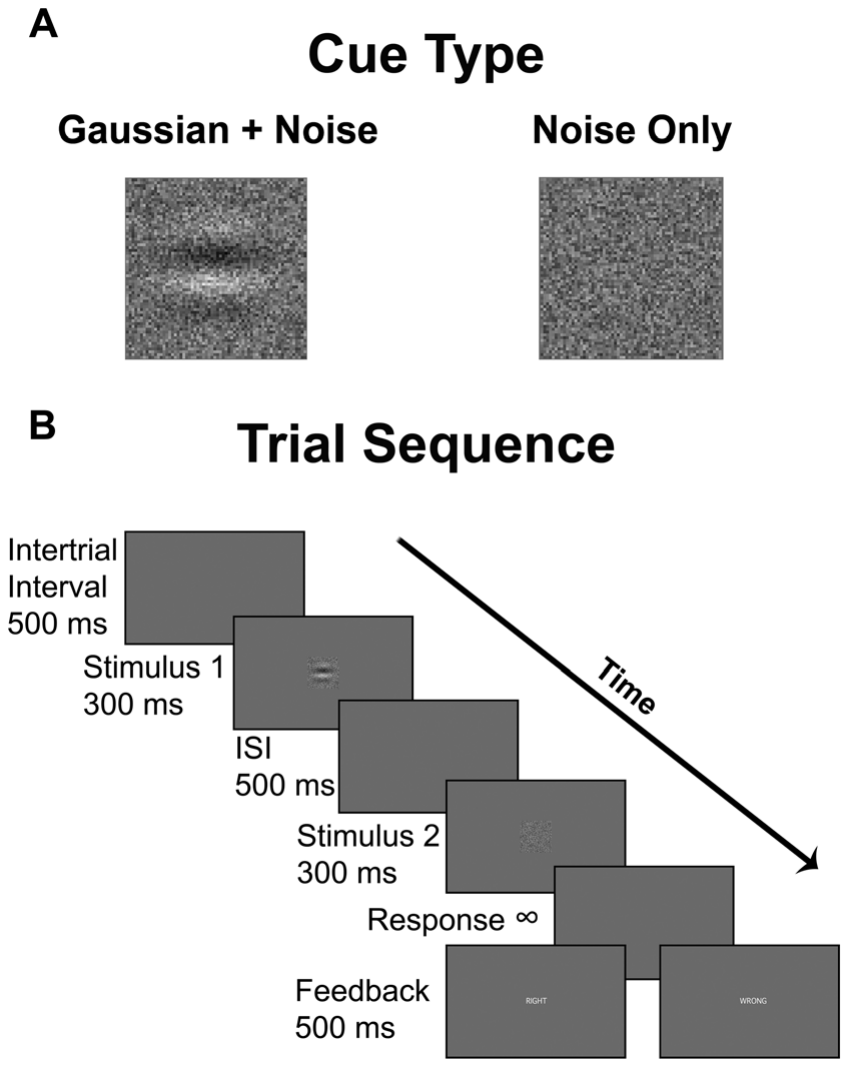
Contrast threshold task. A) Illustration of cue types. Gaussian + Noise example represents the highest contrast stimulus presented on the first trial of the test block. Noise only represents the noise stimulus common to all trials. B) Sequence of events in a trial. In the given example, Stimulus 1 represents the Gaussian + Noise example in 1a and Stimulus 2 represents the Noise only example. Feedback was given in white font as right or wrong based on the accuracy of the participant's response. In the given example, participants would click once to correctly identify the 1st stimulus presented as containing the stimulus with more contrast. ISI  =  inter-stimulus interval.

The task required participants to indicate which of two squares contained a Gaussian grating (described to the participants as a ripple in the visual noise – “as if a single rain drop hit calm water”). Participants were presented with a uniform gray background on which two squares (eye to screen distance ≈60 cm; ≈6.7°×6.7° visual angle) filled with visual noise were presented serially at the central location of the screen. One of the two squares contained a Gaussian grating (approximately 3 cycles per degree, horizontally-oriented) inside the visual noise, while the other contained only noise (Figure 4a). The ratio of signal to noise was determined by QuestMean. Specifically, the grating contrast was manipulated while the noise contrast remained constant. The order of presentation of the square containing the Gaussian grating was randomized. Visual stimuli were presented for 300 ms with an inter-stimulus interval (ISI) of 500 ms. After presentation of the visual stimuli, participants were presented with the uniform gray background until they responded. Participants were required to press the left mouse button once or twice after both visual stimuli had appeared and disappeared from the screen, corresponding to the square containing the Gaussian grating (once for the 1^st^ square, twice for the 2^nd^ square). Following their response, participants received error feedback, followed by an intertrial interval of 500 ms before the next pair of stimuli was presented (see Figure 4b). As the trials progressed, the discriminability of stimulus pairs became more difficult (as determined by the QuestMean algorithm).

#### Contrast discrimination procedure

Participants underwent three blocks of practice trials containing 10 trials each to become accustomed to the procedure. Following practice, participants were administered a block of 80 test trials (Baseline testing). Following Baseline testing, participants underwent the stimulation procedure appropriate to their group. Immediately following stimulation, participants completed another block of 80 test trials (Post-Stimulation testing). Contrast threshold was defined as the value of contrast that yielded 82 percent correct performance across the 80 trials. [42]–[43]

#### Analyses

To assess the effects of cold pressor stimulation (CPS) on contrast thresholds we performed a 2 (Group: Arousal vs. Sham) x 2 (Block: Baseline vs. Stimulation) repeated measures analysis of variance (RM-ANOVA). If CPS influenced sensitivity to contrast in the Arousal Stimulation group, we would expect to find a significant Group x Block interaction in the RM-ANOVAs.

### Results

**Figure 5:**
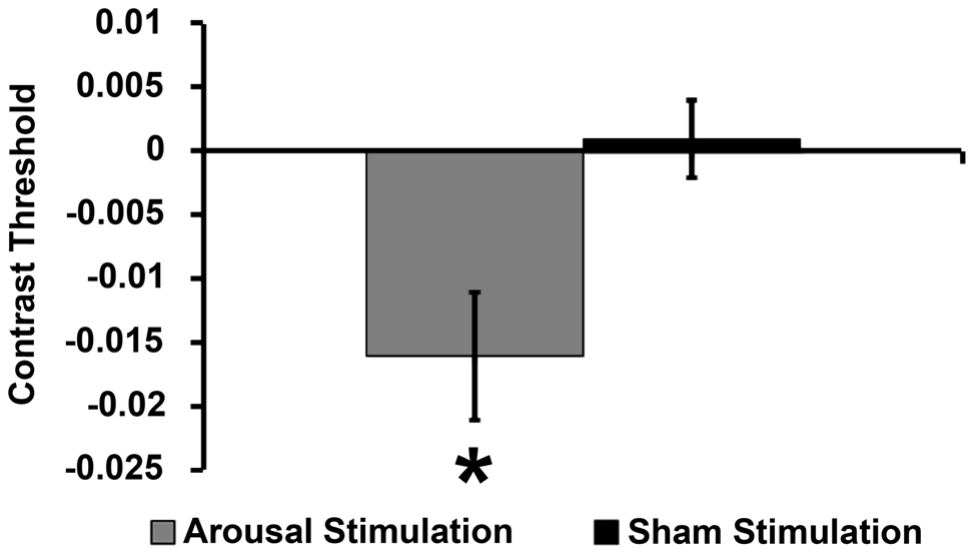
Contrast thresholds. Mean contrast threshold from Baseline to Post-Stimulation for the Arousal Group and Sham Control Group. Baseline performance was not significantly different between groups (t = 1.29; DF = 32; p = .21). * = p<.05

**Figure 6:**
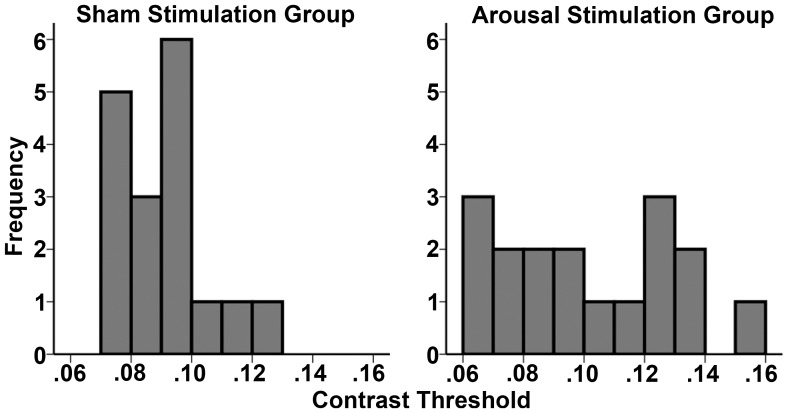
Frequency plots for baseline contrast thresholds by group.

Results for contrast thresholds demonstrated a significant Group x Block interaction (F_1,16_ = 5.24, *p* = 0.03, Mean Square Error (MSE) = 0.001, ηp^2^ = 0.25). Neither Group (F_1,16_ = 0.06, *p* = 0.8) nor Block (F_1,16_ = 3.92, *p* = 0.07) were significant in the RM-ANOVA model. Planned contrasts utilizing paired-samples t-test between Baseline (mean threshold ± standard error (SE) = 0.102±0.006) and Stimulation (mean threshold ± standard error (M±SE) = 0.086±0.004) for each group demonstrated a significant effect of CPS on contrast thresholds from Baseline to Stimulation for participants in the Arousal group (*t* = 2.38, DF = 16, *p* = 0.03, Cohen's *d* = 0.84; Figure 5. Performance from Baseline (M±SE = 0.092±0.003) to Stimulation (M±SE = 0.093±0.003) in the Sham Stimulation group was not significantly different (*t* = −0.07, DF = 16, *p* = 0.9). As is evidenced from the planned contrasts, the marginal significance of Block was a result of the large difference from Baseline to Stimulation in the Arousal group. There was not a significant difference between baseline contrast thresholds in the Arousal versus Sham stimulation groups (independent t-test: t = 1.29; DF = 32; p = .21; Figure 5). Nevertheless, frequency plots of baseline thresholds for the Arousal and Sham Stimulation Groups demonstrate some differences in the distribution of threshold values at baseline (Figure 6). To determine whether non-significant differences at baseline between groups account for significant effects of Arousal Stimulation, we performed an analysis of covariance (ANCOVA) with signed difference in threshold (post-stimulation - baseline) as the dependent variable with Group and Baseline as independent factors. When controlling for differences in baseline thresholds between groups, there remained a significant effect of Group (F_(1,30)_ = 4.6, MSE = .001, p = .03). Thus, slight numerical differences at baseline between Arousal and Sham Stimulation groups do not explain significant effects of CPS on contrast thresholds.

### Discussion

Participants contrast thresholds significantly decreased following CPS. There was no change in participant's contrast thresholds following room temperature sham stimulation. Consistent effects across stereoacuity and contrast thresholds suggest that hyper-arousal has a relatively broad impact on detection of subtle visual differences in the environment. This pattern of result provides strong evidence supporting future investigations into the role of arousal in other aspects of visual perception. Furthermore, these data suggest that a sustained and generalized manipulation of arousal produces similar decreases in thresholds found using transient cued attention to a spatial location and transient manipulations of emotional arousal. As many factors can produce sustained changes in arousal, these findings have broad practical and scientific implications.

### General discussion and conclusions

The present research presents the first evidence that hyper-arousal from sensory stimulation can influence aspects of human visual perception. Participants' sensitivity to subtle depth and contrast differences increased after exposure to CPS, but not after sham stimulation. Decreased visual thresholds following a sustained and generalized manipulation of arousal has a number of important implications. First, this suggests that visual perceptual processing of depth and contrast, and perhaps judgments of other visual relationships, might vary systematically depending upon a variety of factors, including time of day, health status, food consumption, drug history, anxiety, sleep history, etc. These effects could play a role in enhancing survival, driving safety, sports performance, and so on.

Second, understanding the scope and origin of hyper-arousal effects following CPS is an important next step in identifying its applicability to real-world tasks. In addition to depth processing, hyper-arousal could influence other aspects of visual perception, other types of sensory perception, or even non-sensory processes. Future research evaluating these possibilities will help to establish the breadth of hyper-arousal's effect and future directions of its application. For example, Woods and colleagues (2012) recently demonstrated that CPS arousal stimulation temporarily ameliorated inattention and sensory magnitude estimation symptoms of unilateral spatial neglect and normalized the P50 ERP biomarker of arousal in a patient with chronic right-hemisphere stroke. [3] Furthermore, in acute clinical settings, physicians use drugs like modafinil to treat arousal-related deficits following brain injury. [10] Better understanding of the perceptual and cognitive effects of hyper-arousal will inform efforts to identify other populations that might benefit from such treatment and the brain mechanisms that underlie clinical improvement.

Our results also motivate the need to understand what psychological and physiological factors are impacted when behavioral performance is influenced by hyper-arousal. When depth thresholds are reduced by hyper-arousal, for example, is this because depth intervals appear slightly larger due to facilitation of attentional processes? The reciprocal relationship between attention and arousal, as described by Carrasco and colleagues as well as the emotional arousal literature [31]–[33], [35], [38]–[40], provide strong empirical and theoretical foundations for investigating attentional mechanisms in hyper-arousal-related effects. Furthermore, significantly decreased visual thresholds by a sustained and generalized manipulation of arousal provide an important starting point for future investigations into the relationship between attention and arousal. Research demonstrating that stereoacuity improves as a function of stimulus contrast also provides a plausible avenue for future investigations into the underlying mechanisms of arousal-related improvement in visual perception. [45] Specifically, this research could suggest that arousal-related improvements in contrast sensitivity serve as the mechanism underlying changes in stereoacuity thresholds in the present study. If true, other aspects of visual perception modulated contrast sensitivity may also benefit from hyper-arousal. Progress in each of these domains will help identify other tasks that stand to benefit most from stimulation. It will also help generate evidence-based predictions for other aspects of perception and cognition potentially influenced by hyper-arousal. As the responsiveness of neural systems is strongly modulated by arousal state [29]–[30], [46]–[48], a better understanding of the behavioral and neural mechanisms of brain arousal systems will have far reaching implications for multiple fields of science.

In summary, arousal serves as an underlying component for human cognition and consciousness. Hypo-arousal can compromise a broad range of behaviors. In contrast, the present research demonstrates that induction of a hyper-arousal response can decrease visual thresholds. Hyper-arousal effects may also extend to many other components of human behavior. As many factors influence our arousal state, understanding its role in human behavior will be an important area of investigation for future research.
